# Fibroid Tumor of Shoulder

**Published:** 1890-04

**Authors:** Williamson Bryden

**Affiliations:** Boston, Mass.


### FIBROID TUMOR OF SHOULDER.

By Williamson Bryden, V.S.,
Boston, Mass.

On January 23d, 1890, I was called to the stable of William T. Van Nostrand & Co., brewers, at Charleston Neck. A large buckskin colored horse, 12 years old, and weighing about 1,400 pounds, was laid up on account of lameness in the off hind foot, and an enlargement near the point of the off shoulder as large as a child's head which had not been noticed till the morning I first called, when I found it very hard but not very painful.

The hind foot had to be poulticed and pared out. He was re-shod on the 27th, and put to light work on the 28th for half a day, *with the same collar on*. This was continued for five days, at the end of which the enlargement was ready to open. This was done and about a pint of matter escaped. On the 12th of February the enlargement had entirely disappeared, and only a small wound and trifling discharge remained to show where it had been; he has been at work since. Had it not been for the lame hind foot which prevented his being used for five days it would have been well in much less than three weeks. I have treated these injuries from the collar in this way for the last four or five years and have had no trouble in curing them. I usually dress them with warm carbolic solution. The formation of pus is a second stage of the injury. If the horse is kept at work with the same collar for a sufficient length of time, say two or three days, matter can always be found, but if laid off work as soon as detected it may not be present.

